# Bioinformatics analysis revealed the potential crosstalk genes and molecular mechanisms between intracranial aneurysms and periodontitis

**DOI:** 10.1186/s12920-024-01864-0

**Published:** 2024-04-29

**Authors:** Yao Chen, Jian-huang Huang, Yuan-bao Kang, Zheng-jian Yao, Jian-hua Song

**Affiliations:** Department of Neurosurgery, Affiliated Hospital of Putian University, Putian, Fujian Province China

**Keywords:** Intracranial aneurysms, Periodontitis, Crosstalk genes, Immune infiltration, Bioinformatics analysis

## Abstract

**Objectives:**

The risk of intracranial aneurysms (IAs) development and rupture is significantly higher in patients with periodontitis (PD), suggesting an association between the two. However, the specific mechanisms of association between these two diseases have not been fully investigated.

**Materials and methods:**

In this study, we downloaded IAs and PD data from the Gene Expression Omnibus. Differentially expressed genes (DEGs) were identified, and functional enrichment analysis was performed. The protein-protein interaction (PPI) network and weighted gene co-expression network analysis (WGCNA) was performed to identified key modules and key crosstalk genes. In addition, the immune cell landscape was assessed and the correlation of key crosstalk genes with each immune cell was calculated. Finally, transcription factors (TFs) regulating key crosstalk genes were explored.

**Results:**

127 overlapping DEGs were identified and functional enrichment analysis highlighted the important role of immune reflection in the pathogenesis of IAs and PD. We identified ITGAX and COL4A2 as key crosstalk genes. In addition, the expression of multiple immune cells was significantly elevated in PDs and IAs compared to controls, and both key crosstalk genes were significantly negatively associated with Macrophages M2. Finally, GATA2 was identified as a potential key transcription factor (TF), which regulates two key crosstalk gene.

**Conclusions:**

The present study identifies key crosstalk genes and TF in PD and IAs, providing new insights for further study of the co-pathogenesis of PD and IAs from an immune and inflammatory perspective. Also, this is the first study to report the above findings.

**Supplementary Information:**

The online version contains supplementary material available at 10.1186/s12920-024-01864-0.

## Introduction

Intracranial aneurysms (IAs) are a common neurosurgical condition and a major cause of nontraumatic subarachnoid hemorrhage [1]. The prevalence in adults is 2–6%, and the pathology is characterized by local structural deterioration of the arterial wall with loss of the internal elastic layer and media rupture[2, 3]. IAs is an inflammatory disease with both genetic and environmental risk factors [4]. The former is mainly familial IAs, which are less common [5]; the vast majority of IAs are sporadic, and susceptibility factors include hypertension and smoking [6]. The consequences of a ruptured intracranial aneurysms can be catastrophic. A study showed that the risk of death at 5, 10 and 15 years after subarachnoid hemorrhage was 12.9%, 23.6% and 35.4%, respectively [7]. Therefore, research into the pathogenesis of intracranial aneurysms, especially the prevention of unruptured intracranial aneurysmsand improved detection of patients with unruptured intracranialaneurysms is essential to avoid this type of hemorrhagic stroke.

Periodontitis (PD) is a chronic inflammation of the periodontal tissue caused mainly by eriodontal pathogens, can lead to irreversible destruction of tooth-supporting tissues (gums, periodontal ligaments and alveolar bone) [8]with an overall prevalence of 45-50% [9]. Periodontal pathogens include Porphyromonas gingivalis (P. gingivalis), tannerella lengae and dense spirochetes [10]. A large number of periodontal pathogens are present in the deep periodontal pockets of patients with chronic periodontitis, some of which enter the bloodstream, leading to persistent bacteremia and the release of large amounts of inflammatory mediators that cause low-level systemic inflammation, factors that can affect and lead to organ disease away from the mouth [11]. There is now substantial evidence to support an independent association between severe periodontitis and several non-communicable diseases, including diabetes, cerebrovascular disease, and cancers of the digestive tract[12, 13, 14]. To date, more and more evidence suggests that an association between IAs and PD. First, IAs share a high degree of risk factors with PD, including smoking and obesity [15]. Second, multiple case-control studies of patients with IAs have shown that periodontitis is independently and positively associated with the risk of IAs and aneurysmal subarachnoid hemorrhage (aSAH)[16, 17]. For example, periodontitis (≥ 4 mm gingival pockets) and severe periodontitis (≥ 6 mm gingival pockets) were seen in 92% and 49% of patients with IAs, respectively, and were associated with IAs (OR 5.3, 95% CI 1.1–25.9, *p* < 0.000; OR 6.3, 95% CI 1.3–31.4, *p* < 0.001) [16]. Another longitudinal study of the relationship between unruptured intracranialaneurysms formation and periodontal disease showed that the presence of periodontal disease was significantly associated with an increased risk of unruptured intracranial aneurysms [18].

The current view is that IAs are the end result of high-flow blood flow exerting high shear forces and flow-driven inflammatory cell-mediated remodeling of the intracranial arterial wall [19]. Up to 50% of IAs have DNA of oral pathogens in the aneurysmal wall [20], such as P. gingivalis, the most important causative agent of periodontal infections [10].P. gingivalis is a gram-negative bacterium, which lurks in periodontal pockets (worsening the periodontal condition), but is also capable of increased risk of myocardial infarction. In a cross-sectional study of heart attacks, intracoronary thrombosis was found to be associated with P. gingivalis [21]. Animal study also have shown that P. gingivalis can break the blood-brain barrier in several ways, activating microglia to produce a chronic pro-inflammatory environment that is important for the development of Alzheimer’s disease [22].P. gingivalis can remain viable in human macrophages and dendritic cells and may propagate to the aneurysmal wall of IAs through macrophage infiltration, which is strongly associated with inflammatory remodeling of IAs [23]. In addition, exposure to periodontal pathogens and immune response secondary to dysfunction may cause an increased risk of IAs formation and rupture in patients with periodontal disease [24]. These findings suggest a strong association between IAs and PD, but the specific molecular mechanisms and pathological interactions are not fully understood.

In this study, we utilized publicly available datasets from the GEO database, including the test datasets GSE54083 [IAs] and GSE10334 [PD] and the validation datasets GSE75436 [IAs] and GSE16134 [PD]. We used a combination of differential analysis, weighted correlation network analysis (WGCNA) and immune infiltration analysis to confirm that ITGAX and COL4A2 genes play an important role in the common pathogenesis of IAs and PD. Thus, our study may provide new clues to the co-pathogenesis of IAs and PD.

## Materials and methods

### Data source

**Fig. 1:**
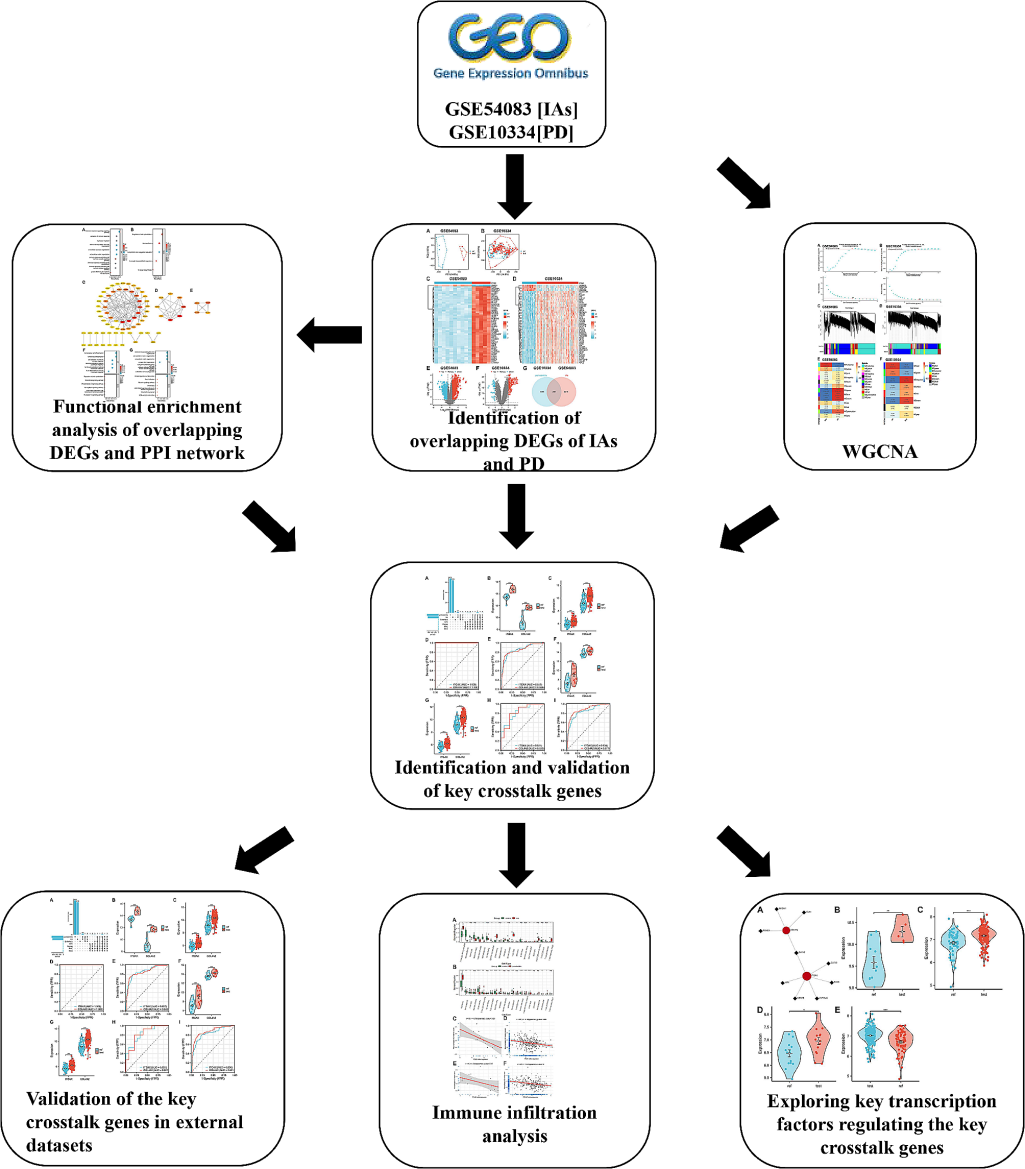
The flow diagram for the whole study

First we show the flow chart of this study (Fig. 1). The expression data of IAs and PDs were obtained from the GEO data base (https://www.ncbi.nlm.nih.gov/geo). The search strategy for this study included: (1) subject searches for “intracranial aneurysms” and “periodontitis”, respectively; (2) Study type option selection “Expression profiling by array”; (3) samples were obtained from Homo sapiens; (4) the dataset contained normal control group samples. The mRNA sequencing of the data set GSE54083 was based on GPL4133 Agilent-014850 Whole Human Genome Microarray 4 × 44 K G4112F (Feature Number version). The mRNA sequencing of GSE10334 samples was based on GPL570 [HG-U133_Plus_2] Affymetrix Human Genome U133 Plus 2.0. The former includes 8 ruptured intracranial aneurysms samples, 5 unruptured aneurysm samples and 10 Superficial temporal artery samples, but the ruptured intracranial aneurysms samples will be excluded in this study. The latter contains 183 PD-affected gingival tissue samples and 64 unaffected gingival tissue samples.

### Differentially expression analysis

Our preliminary analyses of the dataset were based on R software (version 4.2.1; https://cran.r-project.org). Before conducting data analysis, we performed data cleaning, which included normalization using the “normalizeBetweenArrays” function and log2 transformation. DEGs were identified by the"Limma” package [25]. The genes with |log2 fold change| > 0.5 and adjusted P-value < 0.05 were identified as DEGs. Genes that were both up-regulated or down-regulated in both sets of DEGs were defined as overlapping DEGs. Veen plots of overlapping DEGs were plotted with the help of the “ggvenn” package.

### Enrichment analysis of overlapping DEGs

We used the “clusterProfiler” package to perform enrichment analysis of overlapping DEGs, including Gene Ontology Biological Process (GO_BP) enrichment analysis [26]. Kyoto Encyclopedia of Genes and Genomes (KEGG) pathway enrichment analysis [27].Terms with FDR < 0.05 were considered to be significantly enriched.

### Protein-protein interaction (PPI) network construction

We use the STRING database V11.5 (http://string-db.org) to build a PPI network with overlapping DEGs and set the “minimum required interaction score” parameter to 0.4 to hide the unconnected nodes. We obtained the interaction score of the overlapping DEGs, which was then imported the Cytoscape software V3.9.1 [28]for visualization. The MCODE plug in was used to filter clusters with high connectivity, thus dividing the PPI network into multiple clusters with default parameters. Finally, the genes in the clusters were analyzed for functional enrichment.

### Weighted gene co-expression network analysis (WGCNA)

The WGCNA analysis can identify genes with high correlation, i.e., genes that are grouped into modules with similar functions, and can perform correlation analysis between the modules and phenotypic data to discover potential key genes. The top 5000 genes with the highest absolute median difference in expression in the test data set were screened for WGCNA analysis using the “WGCNA” package [29].First, a soft threshold was obtained using the pickSoftThreshold function. Then, a weighted adjacency matrix was constructed. Finally, the correlation between each module and the disease was calculated. The module with the highest correlation with the grouped traits was defined as the key module. The genes within the key module were correlated with the grouped traits.

### Identification and validation of key crosstalk genes

First, we use the CytoHubba plugin of Cytoscape software to filter the hub genes in the PPI network [30]. The genes that were ranked in the top 20 using different algorithms [Maximal Clique Centrality (MCC); Maximum Neighborhood Component (MNC); Edge Percolated Component (EPC) ] were identified as PPI key genes [30]. Next, the PPI key genes were intersected with the key module genes of WGCNA, and these intersected genes were defined as key crosstalk genes. Subsequently, we compared the mRNA expression levels of key crosstalk genes between the case and control groups using the Mann-Whitney U test, and *P* < 0.05 was considered statistically significant, and visualized by “ggplot2”. Finally, we tested the diagnostic efficacy of key crosstalk genes in the test dataset using the receiver operating characteristic curves (ROCs) of the “pROC” package.

### Validation of key crosstalk genes in an independent external dataset

When selecting an external validation dataset, we followed the same screening principles to minimize bias. We validated the mRNA expression levels of key crosstalk genes in independent external datasets GSE75436 and GSE16134 samples. GSE75436 included 15 normal superficial temporal artery samples, 15 intracranial aneurysms samples, and mRNA sequencing of the samples was based on GPL570 [HG-U133_Plus_2] Affymetrix Human Genome U133 Plus 2.0 Array. GSE16134 contains 241 PD-affected gingival tissue samples and 69 unaffected gingival tissue samples, mRNA sequencing of the samples is based on GPL570 [HG-U133_Plus_2] Affymetrix Human Genome U133 Plus 2.0 Array.

### Immuno-infiltration analysis

First, immune cell expression levels in the test dataset were analyzed in the case and control groups using the “cibersort” algorithm [31]. We then calculated the Pearson correlation coefficient and P-value between the expression of key genes that cause cross-talk and the expression of immune cells., *P* < 0.05 was considered statistically significant and was visualized by “ggplot2”.

### Identification of transcription factors (TFs) of key crosstalk genes

TFs of key crosstalk genes were predicted by NetworkAnalyst 3.0 (https://www.networkanalyst.ca) [32]. Subsequently, we compared the average expression levels of TFs in the test set and validation set samples using the Mann-Whitney U test. Finally, TFs that were typically upregulated in the case set were identified as potential key TFs in IAs and PDs.

## Results

### Identify overlapping DEGs of IAs and PDs

**Fig. 2:**
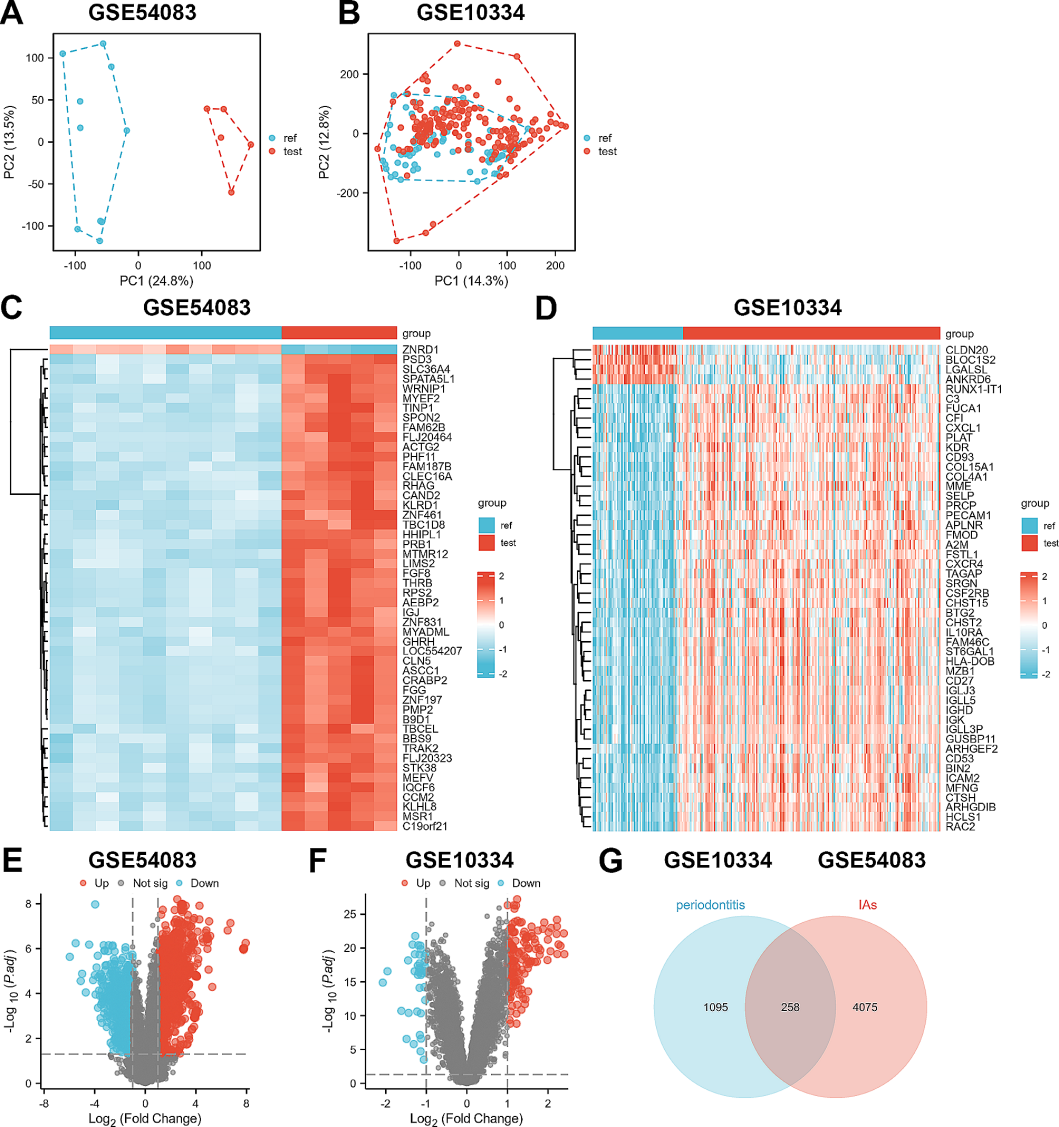
DEGs of test datasets GSE54083 and GSE10334. (**A**), (**C**), and (**E**) are PCA plots, heat maps, and volcano plots of GSE54083 DEG analysis, respectively. (**B**), (**D**), and (**F**) are PCA plots, heat maps, and volcano plots of GSE10334 DEG analysis, respectively. (**G**). DEGs of GSE54083 and GSE10334 are crossed to obtain 258 intersecting DEGs

The “PCA” function was used to downscale the high-latitude data, and significant sample differences were found between the test and control groups in the two datasets (Fig. 2A-D). In the dataset GSE54083, the “Limma” R package identified 4333 DEGs, of which 2364 were up-regulated and 1969 were down-regulated (Fig. 2E). In the dataset GSE10334, the “Limma” R package identified 1353 DEGs, of which 813 were up-regulated and 540 were down-regulated (Fig. 2F). Subsequently, we identified 258 intersecting DEGs (Fig. 2G). Finally, the above genes were examined for trend checked and 127 overlapping DEGs were obtained (S1).

### Functional enrichment analysis of overlapping DEGs

**Fig. 3:**
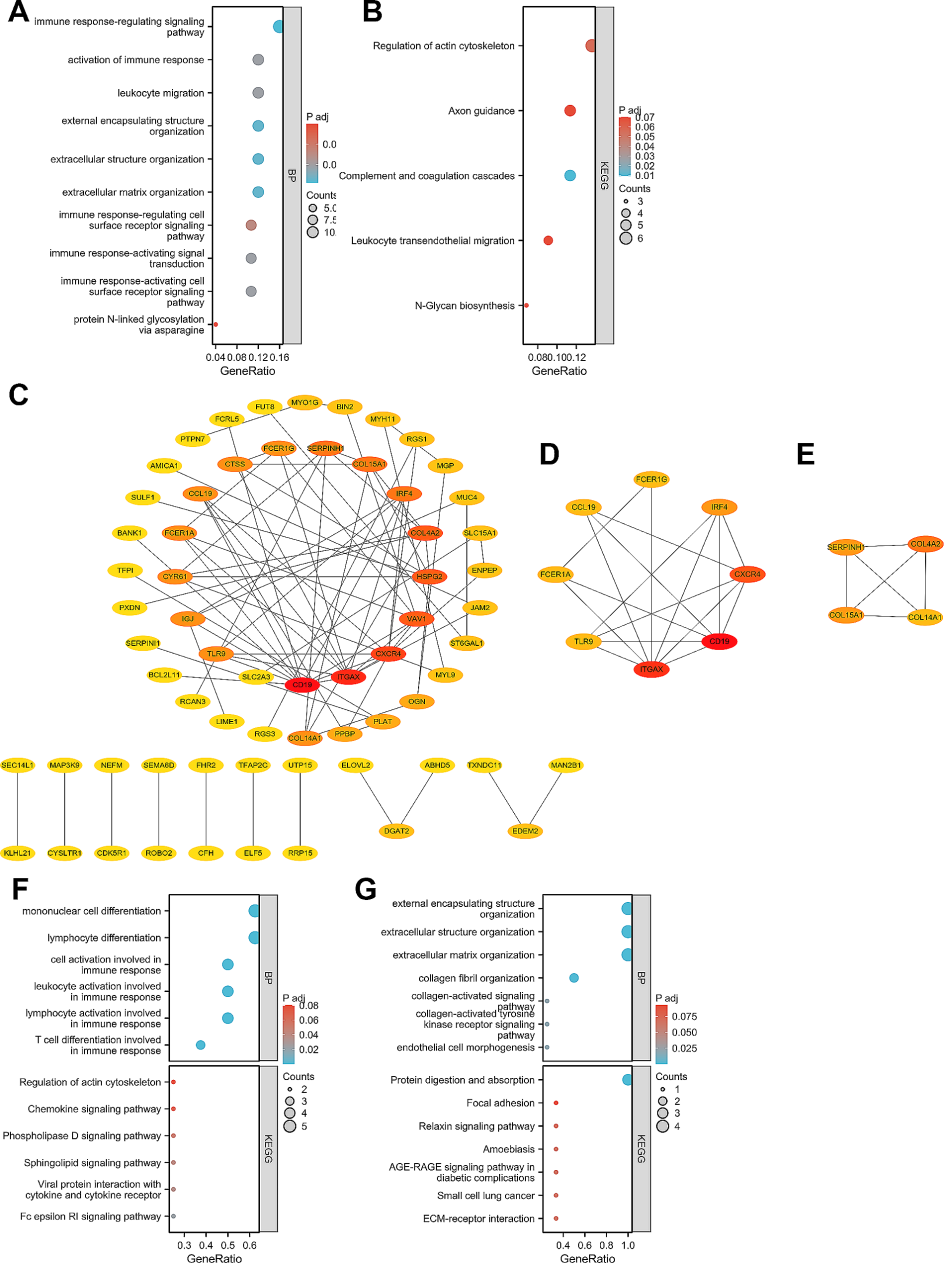
Functional enrichment analysis and PPI network construction for overlapping DEGs of the test dataset. (**A**) GP-BP enrichment analysis of overlapping DEGs. (**B**) KEGG enrichment analysis of overlapping DEGs. (**C**) PPI network constructed for overlapping DEGs. (**D**-**E**). PPI network obtained by the Mcode algorithm for cluster 1 and cluster 2. (**F**-**G**). GP-BP enrichment analysis and KEGG enrichment analysis of genes contained in cluster 1 and cluster 2. enrichment analysis and KEGG enrichment analysis

The GO_BP analysis showed that the most significantly enriched terms were immune response-regulating signaling pathway, extracellular matrix organization, extracellular structure organization, external encapsulating structure organization, leukocyte migration, and activation of immune response (Fig. 3A). kEGG analysis showed that overlapping DEGs may be associated with Regulation of actin cytoskeleton, Axon guidance, Complement and coagulation cascades, and Leukocyte transendothelial migration (Fig. 3B). Thus, overlapping DEG functions are clearly associated with immune and inflammatory processes.

### Building PPI networks with overlapping DEGs

Overlapping DEGs were entered into the STRING database and a PPI network with 81 nodes and 123 edges (PPI enrichment p-value < 4.33e-09) was constructed at a medium confidence level (0.4). The PPI network was visualized using Cytoscape software (Fig. 3C). Two clusters with high connectivity were identified by the Mcode plug-in (Fig. 3D, E). Cluster 1 contains 8 nodes and 17 edges; Cluster 2 contains 4 nodes and 6 edges. Subsequently, we performed GP-BP enrichment analysis and KEGG enrichment analysis on the genes contained in Cluster 1 and Cluster 2 (Fig. 3F, G). The functional enrichment of GO-BP for cluster 1 intronic genes mainly included lymphocyte differentiation, mononuclear cell differentiation, lymphocyte activation involved in immune response, leukocyte activation involved in immune response and T cell differentiation involved in immune response, etc. KEGG enrichment analysis for cluster 1 intronic gene mainly includes Fc epsilon RI signaling pathway, Viral protein interaction with cytokine and cytokine receptor and Sphingolipid signaling pathway, etc. The functional enrichment of GO-BP for cluster 2 intronic genes mainly included extracellular matrix organization, extracellular structure organization, external encapsulating structure organization, collagen fibril organization and collagen-activated signaling pathwa, etc. KEGG enrichment analysis for cluster 2 intronic genemainly included ECM-receptor interaction, AGE-RAGE signaling pathway in diabetic complications, Amoebiasis, Relaxin signaling pathway and Focal adhesion, etc.

### Weighted gene co-expression network construction and key module screening

**Fig. 4:**
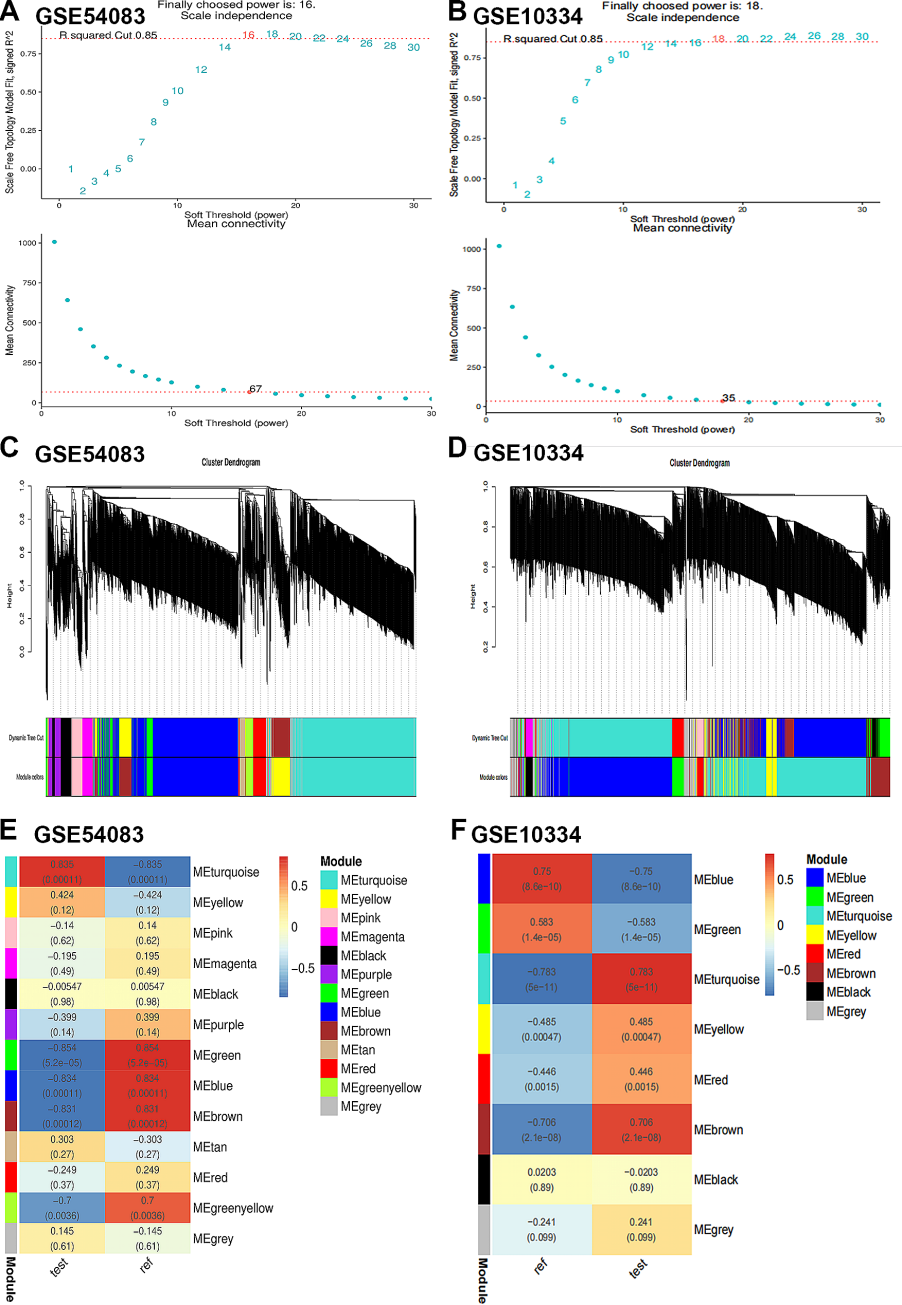
Weighted gene co-expression network analysis (WGCNA) of the test dataset. (**A**-**B**). Soft threshold selection for GSE54083 and GSE10334. (**C**-**D**). Clustering dendrogram of the top 5000 genes with the highest absolute median difference in expression between GSE54083 and GSE10334 based on differential measures. (**E**-**F**). Module-trait relationship between GSE54083 and GSE10334 in relation to the modules and traits. Different colors represent different modules and contain the corresponding correlations and p-values

The top 5000 genes with the highest absolute median difference in expression in the test dataset were screened for WGCNA analysis. β = 16 and β = 18 (scale-free R2 = 0.85) were chosen as soft thresholds for IAs and PDs, respectively, to ensure scale-free networks (Fig. 4A and B). 13 modules were identified in GSE54083 and 8 modules in GSE10334 by WGCNA analysis (Fig. 4C and D). Finally, a heat map of module-trait relationships was generated based on Pearson correlation coefficients. The results showed that the green module was the most correlated with IAs (0.854, *p* = 5.2E-05) and contained 661 genes; the turquoise module was the most correlated with PD (0.783, *p* = 5E-11) and included 705 genes (Fig. 4E and F).

### Identification and validation of key crosstalk genes

**Fig. 5:**
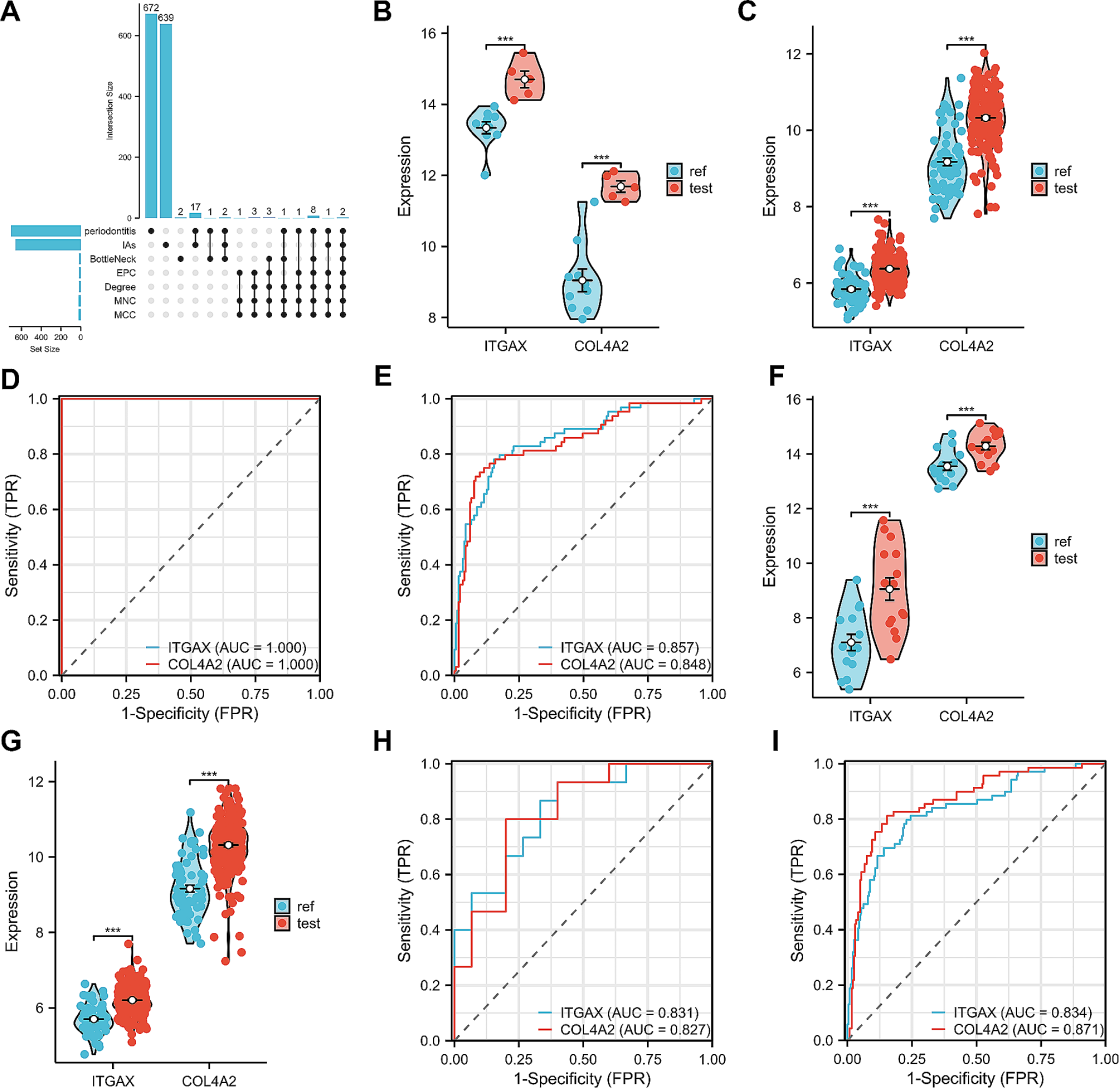
Identification and validation of key crosstalk genes. (**A**). ITGAX and COL4A2 were both present in the four algorithms and two WGCNA key modules. (**B**-**C**). ITGAX and COL4A2 expression levels were higher in the case group than in the control group in GSE54083 and GSE10334. (**D**-**E**). ITGAX and COL4A2 had good diagnostic capabilities in GSE54083 and GSE10334. (**F**-**G**). The expression levels of ITGAX and COL4A2 were higher in the GSE75436 and GSE16134 case groups than in the control group. (**H-I**). ITGAX and COL4A2 had good diagnostic capabilities in GSE75436 and GSE16134. (***, *p* < 0.0001)

We used the CytoHubba plugin to sort the top 20 genes under four algorithms (Degree, EPC, MCC and MNC). The Upset plot showed that ITGAX and COL4A2 were present in both the top 20 genes of the four algorithms and in the two key modules (Fig. 5A). Therefore, these two genes were identified as key crosstalk genes. In addition, the ITGAX and COL4A2 mRNA expression levels were significantly higher in the case group than in the control group in GSE54083 and GSE10334 (Fig. 5B, C). In addition, ITGAX and COL4A2 had good diagnostic ability for IAs and PD according to ROC curves. The AUC values of ITGAX in GSE54083 and GSE10334 were 1.000 and 0.857, respectively. And the AUC values of COL4A2in GSE54083 and GSE10334 were 1.000 and 0.848, respectively (Fig. 5D, E).

### Validation of key crosstalk genes in an independent external datasets

To increase the confidence level, we validated the expression of key crosstalk genes in two additional independent external datasets (GSE75436 and GSE16134). Consistent with the results of the test set, ITGAX and COL4A2 mRNA expression levels were elevated in the case set (Fig. 5F, G), while showing good diagnostic efficiency. The AUC values of ITGAX in GSE75436 and GSE16134 were 0.831 and 0.834, respectively. And the AUC values of COL4A2in GSE75436 and GSE16134 were 0.827 and 0.871, respectively (Fig. 5H, I).

### Immuno-infiltration analysis

**Fig. 6:**
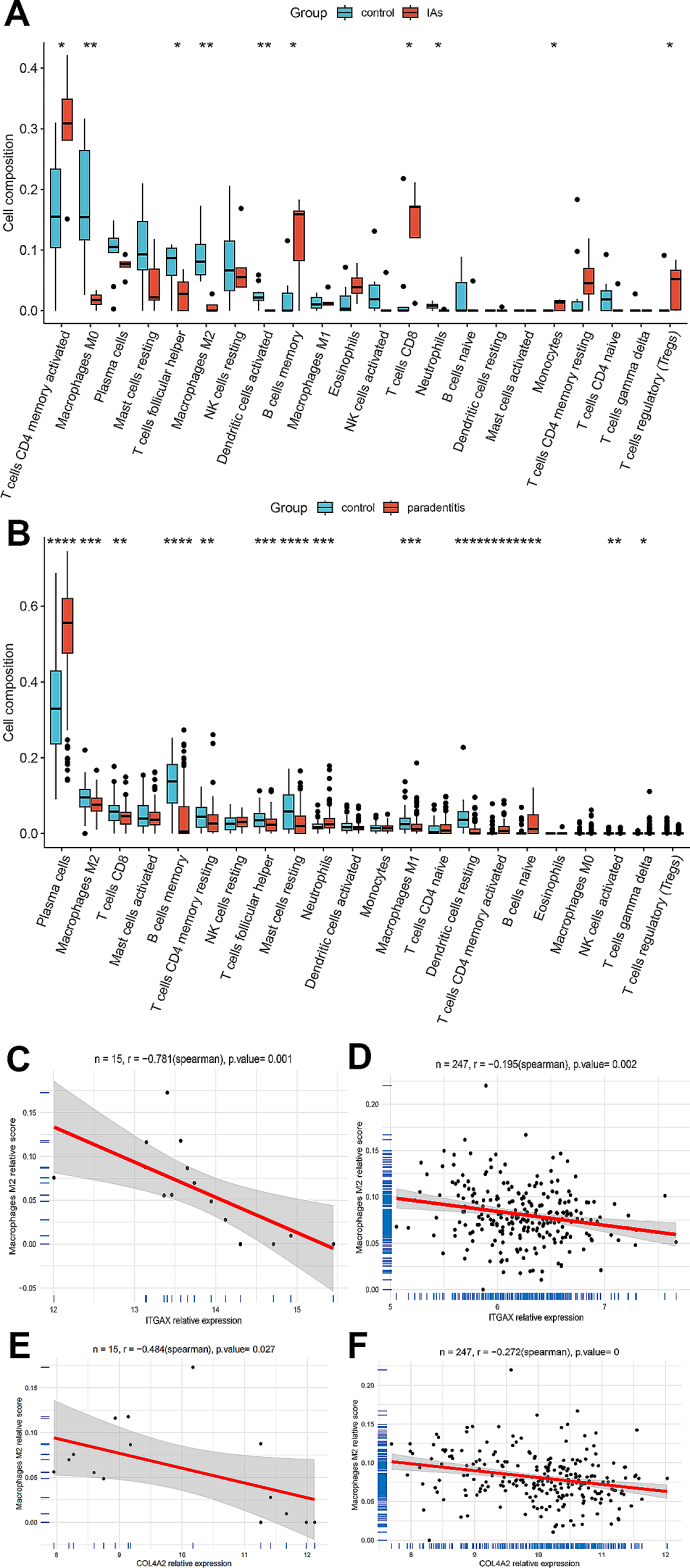
Immune infiltration analysis of the test dataset. (**A**-**B**). The immune landscape was significantly altered in the GSE54083 and GSE10334 case groups. (**C**-**D**). ITGAX and COL4A2 expression levels in GSE54083 were significantly negatively correlated with Macrophages M2. (**D**-**F**). ITGAX and COL4A2 expression levels in GSE10334 were significantly negatively correlated with Macrophages M2

By performing enrichment analysis of overlapping DEGs, we found that immune and inflammatory processes are involved in crosstalk between IAs and PD. Therefore, we used the cibersort algorithm to analyze the proportion of immune cells in the case group versus control samples in both test datasets. We found that the immune landscape of IAs and PD was significantly altered in the case group (Fig. 6A and B). Furthermore, correlation analysis showed that ITGAX and COL4A2 expression levels were significantly negatively correlated with Macrophages M2 (Fig. 6C-F).

### Exploring key transcription factors (TFs) that regulate key crosstalk genes

**Fig. 7:**
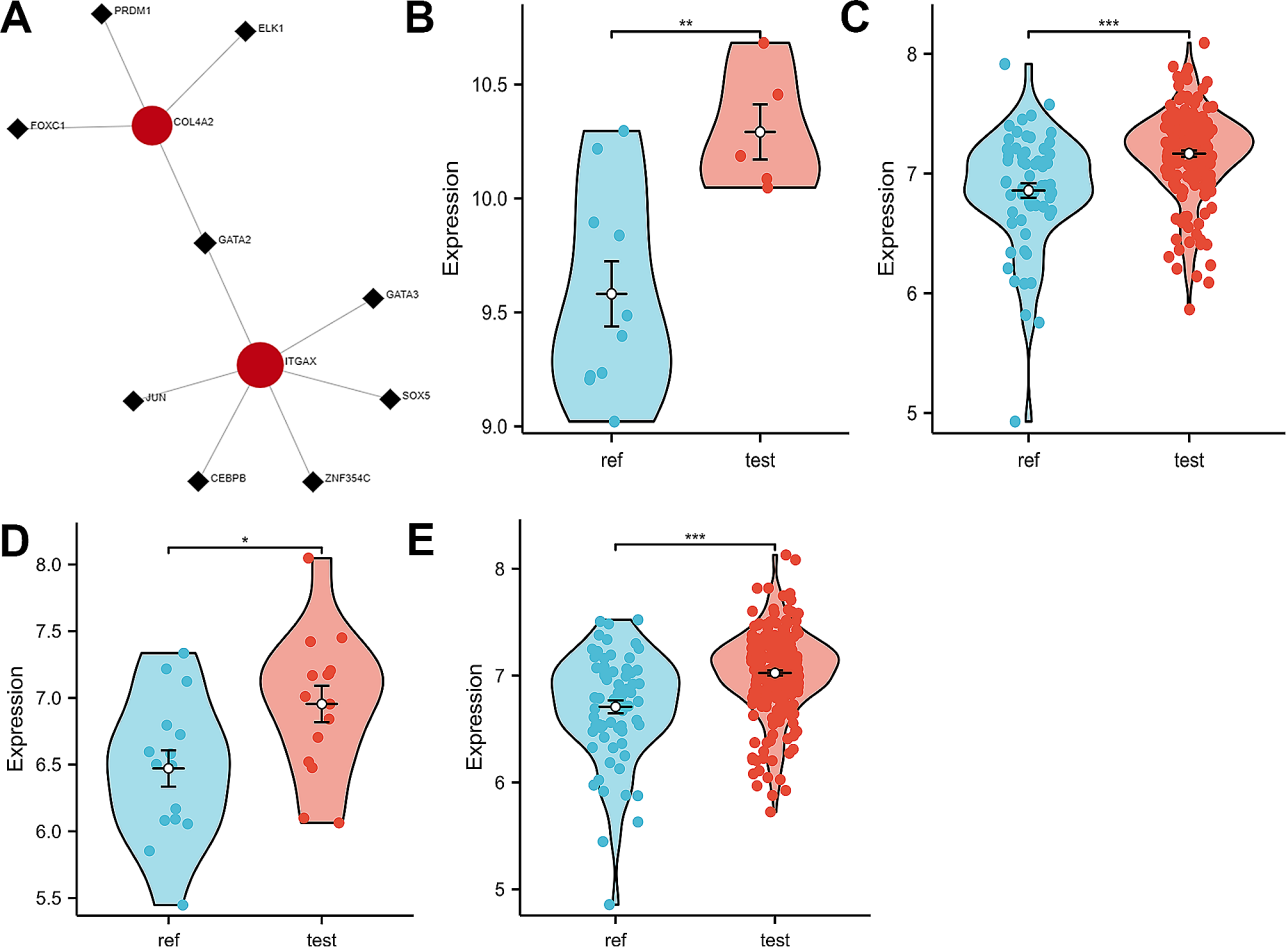
GATA2 was identified as a potential key TF shared in IAs and PD. (**A**). NetworkAnalyst 3.0 suggests that GATA2 regulates both ITGAX and COL4A2. (**B**-**C**). GATA2 was significantly elevated in the test dataset (GSE54083 and GSE10334) case set. (**D-E**). GATA2 was significantly elevated in the external validation dataset (GSE75436 and GSE16134) case group (*, *P* < 0.01; **, *P* < 0.001; ***, *P* < 0.0001)

Further, we explored potential TFs that may regulate ITGAX and COL4A2 genes using NetworkAnalyst 3.0 and compared the expression levels of case and control groups in all datasets by Mann-Whitney U test. We found that GATA2 interacted with both ITGAX and COL4A2 (Fig. 7A), and the expression level of GATA2 was significantly elevated in all case groups (Fig. 7B-E). Thus, GATA2 may be a potential key TF regulating two key crosstalk genes in the pathological process of IAs and PD.

## Discussion

Current studies suggest that periodontitis is not only associated with abdominal aortic aneurysm [33], coronary heart disease [34] and atherosclerosis cardiovascular disease risk [35], but also with the occurrence and rupture of intracranial aneurysms[16, 24].However, the specific mechanisms of the effect of periodontitis on intracranial aneurysms have not been well studied. In the present study, we focused on exploring the key crosstalk genes (ITGAX and COL4A2) shared by intracranial aneurysms and periodontitis as well as the transcription factor (GATA2) regulating key crosstalk genes by bioinformatics approach, which was fully validated in an external independent datasets. To our knowledge, this is the first study to report the above findings.

Through a comprehensive analysis of the datasets, ITGAX and COL4A2 were identified as key crosstalk genes in the comorbidity of IAs and PD. ITGAX, also known as CD11c, complement receptor 4 (CR4), is responsible for encoding integrin αX chain proteins, a marker shared by macrophages and dendritic cells [36].ITGAX can promotes monocyte adhesion and chemotaxis, regulates immune response, and plays an important role in the pathogenesis of infection and atherosclerosis [37]. Macrophages and the inflammatory signals they mediate play an important role in the pathogenesis of IAs[38, 39]. Meanwhile, macrophage levels were significantly higher in periodontitis samples than in healthy samples [40], they are central players in the destruction and repair phases of periodontal disease [41].COL4A2, which encodes the α2 chain of type IV collagen, is a major structural component of basement membranes (BMs) and plays a fundamental and crucial role in vessel wall integrity; Abnormal COL4A2 expression can lead to a broad phenotypic spectrum involving the nervous system, kidneys, and other organs, but the main site of vascular damage is the brain[42, 43]. Compared to familial intracranial aneurysms, abnormal COL4A2 expression in sporadic aneurysms may be associated with aneurysm development[44, 45]. Periodontitis is characterized by irreversible and progressive degeneration of periodontal tissue. Periodontal ligament stem cells (PDLSC) were involved in periodontal tissue regeneration, and COL4A2 in the tissue-specific extracellular matrix plays an important role in this process [46]. Therefore, two upregulated key crosstalk genes may be involved in IAs and PD comorbidity mechanisms.

Immune infiltration plays an important role in the co-pathogenesis of IAs and PD. Crosstalk gene enrichment analysis suggests that immune and inflammatory processes are involved in IAs and PD comorbidity mechanisms. Immune cell infiltration analysis revealed a significantly different immune landscape in IAs and PD disease groups compared to controls, and two key crosstalk genes expression was significantly negatively correlated with Macrophages M2. Several studies in both human and animal models have confirmed the presence of macrophage-dominated immune cell infiltration in IAs and PD tissues. Histopathological analysis of human IAs tissues revealed that the number of macrophage infiltration in ruptured IAs tissues was higher than that in unruptured IAs, suggesting that increased macrophage infiltration in tissues had already occurred prior to the rupture of IAs [47]. Another study in a rat model of IAs suggested that macrophage infiltration in the vessel wall occurs at the time of IAs formation, and that the degree of infiltration increases with the pathological progression of IAs [48]. Thus, macrophage-mediated cellular and molecular inflammation is a key factor in aneurysm formation and rupture [49].At the level of gene transcription, mRNA expression of IL-1β and TNF-α was increased in monocytes isolated from peripheral blood of patients with IAs compared to the healthy population, while IL-10 mRNA did not change significantly, indicating that monocytes showed preferential transcription to M1-type macrophages in patients with IAs [50]. It was also shown that balanced transfer of macrophages to M2 could prevent aneurysm formation and rupture [51]. Similarly, Macrophages play different roles through polarization in different stages of PD [52]. In the early or active phase of periodontitis, macrophage polarization phenotype as M1 type is mainly induced by Th1 production of IFN-γ and LPS-dominated microbial-associated factors [53], while the proportion of M1 is positively correlated with the progression of periodontal inflammatory activity; in the repair phase of PD, M2-associated factor expression, Th2 and Treg cell immunosuppression and repair effects increased [54, 55]. Thus, M1 polarization is a major feature of macrophages in periodontitis and is responsible for the development and progression of periodontal tissue destruction in periodontitis [56].

In summary, we hypothesize the following mechanisms of co-morbidity between IAs and PD. First, periodontal pathogens such as P. gingivalis proliferate in periodontal pockets and release large amounts of inflammatory mediators that recruit large numbers of monocytes and macrophages. P. gingivalis uses complex strategies to evade the major antimicrobial mechanisms of macrophages and inhibit macrophage M2 polarization [57, 58], leading to the onset and progression of periodontitis. In our study, ITGAX was highly expressed and inversely correlated with M2 macrophages. This implies that ITGAX is associated with inhibition of macrophage M2 polarization. Second, when these pathogen-carrying immune cells entered the cerebral arteries with the circulation, underwent transendothelial migration and clustered in the cerebral artery wall [59], matrix metalloproteinases were upregulated, mediated accelerated degradation of type IV collagen, and moreover induced de novo synthesis of new proteins and maintained tissue homeostasis of the basement membrane [60]. COL4A2 encodes the α2 chain of type IV collagen, a major structural component of almost all basement membranes (BMs) and plays a fundamental and critical role in vascular wall integrity. Abnormal COL4A2 expression can lead to a broad spectrum of phenotypes involving the nervous system, kidney, and other organs, but the major site of vascular injury is the brain [42, 43]. Genetic studies have shown that COL4A2 mutations can lead to congenital aneurysms [61]. In addition, COL4A2 is essential for aneurysm development according to a label-free quantitative proteomics study of human intracranial aneurysms [44]. Upregulation of COL4A2 expression may reflect the process of basement membrane remodeling after accelerated degradation of the basement membrane, which is also necessary for the growth of IAs. Therefore, we hypothesize that the immune process mediated by ITGAX and COL4A2 is central to the co-morbid mechanism of intracranial aneurysm and periodontitis.

For further investigate the candidate regulatory mechanisms of key crosstalk genes (ITGAX and COL4A2) shared by intracranial aneurysms and periodontitis, we constructed a target gene-TF network. Our study showed that the potential regulatory network of ITGAX and COL4A2 consists of 9 TFs, in which GATA2 interacts closely with both of these genes. Recent studies have shown that GATA2 may play an important role in regulating the immune response in the mechanism of IAs formation [62]. Meanwhile, phagocytic GATA2 overexpression drives atherosclerosis formation [63, 64]. These findings are consistent with the results of the present study. Currently, no studies have focused on the potential role of GATA2 in the pathogenesis of PD. According to the available data, GATA2 is a zinc-finger transcription factor that is mainly expressed in the hematopoietic system.GATA2 regulates various biological processes to directly or directly influence the progression of atherosclerosis, including aortic neovascularization, hematopoiesis, adipogenesis, and inflammation [65, 66]. For example, it improves monocyte adhesion and promotes leakage through the arterial wall by upregulating VCAM-1 [66].

This study identifies key crosstalk genes and TFs in IAs and PD from an immune and inflammatory perspective, thus providing new insights into the mechanisms of comorbidity in IAs and PD. It is important to note that the conclusion needs more clinical validation in the future. In addition, the specific functions of the crosstalk genes remain to be validated in vivo and in vitro.

## Conclusion

In summary, we identified ITGAX and COL4A2 as key crosstalk genes in IAs and PD by multiple bioinformatics analysis methods, and they may be involved in crosstalk between IAs and PD through immune pathways. In addition, GATA2 was identified as a potential key TF in IAs and PD. To our knowledge, this is the first study to report the above findings. The present study provides new insights into the co-pathogenesis of IAs and PD. This will provide important guidance for the treatment of intracranial aneurysms, particularly the pharmacological treatment and prevention of unruptured aneurysms.

### Electronic supplementary material

Below is the link to the electronic supplementary material.

Supplementary Material 1

Supplementary Material 2

## Data Availability

The datasets generated and analysed during the current study are available in the NCBI Gene Expression Omnibus (GEO) repository, Accession Number GSE54083, GSE10334, GSE75463 and GSE16134.

## Abbreviations

IAs: Intracranial aneurysms
PD: Periodontitis
GEO: Gene Expression Omnibus
DEG: Differentially expressed gene
PPI: Protein-protein Interaction
WGCNA: Weighted Gene Co-expression Network Analysis
GO_BP: Gene Ontology Biological Process
KEGG: Kyoto Encyclopedia of Genes and Genomes
ROCs: Receiver operating characteristic curves
