# Supplementary material for: Bioinformatics analysis revealed the potential crosstalk genes and molecular mechanisms between intracranial aneurysms and periodontitis
Source: BMC Med Genomics. 2024 Apr 29;17:114. doi: 10.1186/s12920-024-01864-0 (PMC11059758; doi:10.1186/s12920-024-01864-0)
Supplement: Supplementary file 2 — Supplementary Material 2 [file 12920_2024_1864_MOESM2_ESM.docx]

####GEO####

install.package('AnnoProbe')

gset=AnnoProbe::geoChina('GSE75436')

gset

suppressMessages(library(GEOquery))

gset=gset[[1]]

phenoDat <- pData(gset)

library(stringr)

group_list <- ifelse(str_detect(phenoDat$title, "temporal artery"), "ref",

"test")

library(stringr)

group_list = factor(group_list,

levels = c("test","ref"))

table(group_list)

exprSet=exprs(gset)

dim(exprSet)

exprSet1 <-exprSet[,-c(1:8)]

exprSet <- exprSet1

boxplot(exprSet,outline=FALSE, notch=T,col=group_list, las=2)

library(limma)

exprSet2=normalizeBetweenArrays(exprSet)

boxplot(exprSet2,outline=FALSE, notch=T,col=group_list, las=2)

dev.off()

ex <- exprSet2

qx <- as.numeric(quantile(ex, c(0., 0.25, 0.5, 0.75, 0.99, 1.0), na.rm=T))

LogC <- (qx[5] > 100) ||

(qx[6]-qx[1] > 50 && qx[2] > 0) ||

(qx[2] > 0 && qx[2] < 1 && qx[4] > 1 && qx[4] < 2)

if (LogC) { ex[which(ex <= 0)] <- NaN

exprSet2 <- log2(ex)

print("log2 transform finished")}else{print("log2 transform not needed")}

library(AnnoProbe)

(gpl=gset@annotation)

ids<-idmap(gpl ,type = 'soft')

exprSet2<-filterEM(exprSet2,ids)

write.table(exprSet2, file = "exp.txt",sep = "\t",row.names = T,col.names = NA,quote = F)

write.table(phenoDat, file = "phenoDat.txt",sep = "\t",row.names = T,col.names = NA,quote = F)

library(limma)

colnames(targets)=c("FileName","Target")

lev<-unique(targets$Target)

f <- factor(targets$Target, levels=lev)

design <- model.matrix(~0+f)

design=model.matrix(~factor(group_list))

fit=lmFit(exprSet2,design)

fit=eBayes(fit)

DEG=topTable(fit,coef=2,n=Inf)

DEG=na.omit(DEG)

write.table(DEG, file = "DEG.txt",sep = "\t",row.names = T,col.names = NA,quote = F)

need_deg=data.frame(symbols=rownames(DEG), logFC=DEG$logFC, p=DEG$P.Value)

deg_volcano(need_deg, style = 1, p_thred = 0.05, logFC_thred = 1)

deg_volcano(need_deg,2)

eg_heatmap(DEG,exprSet2,group_list)

check_diff_genes('ITGAX',exprSet2,group_list)

check_diff_genes('COL4A2',exprSet2,group_list)

gene5 = exprSet2[order(apply(exprSet2,1,mad), decreasing = T)[1:10000],]

write.table(gene5, file = "10000exp.txt",sep = "\t",row.names = T,col.names = NA,quote = F)

X5000GENES <- read_csv("5000GENES.csv")

exprSet$gene <- rownames(exprSet)

datas <- exprSet[which(exprSet$gene %in% X5000GENES$gene),]

write.table(datas, file = "5000exp.txt",sep = "\t",row.names = T,col.names = NA,quote = F)

library(tidyverse)

chooseBioCmirror()

BiocManager::install('GEOquery')

library(GEOquery)

library(limma)

chooseBioCmirror()

gset = getGEO('GSE10334', destdir=".", AnnotGPL = F, getGPL = F)

class(gset)

###

gset[[1]]

pdata <- pData(gset[[1]])

table(pdata$title)

library(stringr)

group_list <- ifelse(str_detect(pdata$title, " Affected site"), "test",

"ref")

group_list = factor(group_list,

levels = c("test","ref"))

exp <- exprs(gset[[1]])

boxplot(exp,outline=FALSE, notch=T,col=group_list, las=2)

dev.off()

range(exp)

exp=normalizeBetweenArrays(exp)

boxplot(exp,outline=FALSE, notch=T,col=group_list, las=2)

range(exp)

dev.off()

index = gset[[1]]@annotation

if(!require("hgu133plus2.db"))

BiocManager::install("hgu133plus2.db")

library(hgu133plus2.db)

ls("package:hgu133plus2.db")

ids <- toTable(hgu133plus2SYMBOL)

head(ids)

#length(unique(ids$symbol))

#table(sort(table(ids$symbol)))

library(tidyverse)

exp <- as.data.frame(exp)

exp <- exp %>% mutate(probe_id=rownames(exp))

exp <- exp %>% inner_join(ids,by="probe_id")

exp <- exp[!duplicated(exp$symbol),]

rownames(exp) <- exp$symbol

exp <- exp[,-(248:249)]

write.table(exp, file = "exp.txt",sep = "\t",row.names = T,col.names = NA,quote = F)

####go+kegg####

library(clusterProfiler)

genes <- read.delim('gene.txt', header = TRUE, stringsAsFactors = FALSE)[[1]]

enrich.go <- enrichGO(gene = genes,

OrgDb = 'homo',

keyType = 'ENTREZID',

ont = 'ALL',

pAdjustMethod = 'fdr',

pvalueCutoff = 0.05,

qvalueCutoff = 0.2,

readable = FALSE)

write.table(enrich.go, 'enrich.go.txt', sep = '\t', row.names = FALSE, quote = FALSE)

genes <- read.delim('gene.txt', header = TRUE, stringsAsFactors = FALSE)[[1]]

kegg <- enrichKEGG(

gene = genes,

keyType = 'kegg',

organism = 'homo',

pAdjustMethod = 'fdr',

pvalueCutoff = 0.05,

qvalueCutoff = 0.2)

write.table(kegg, 'kegg.txt', sep = '\t', quote = FALSE, row.names = FALSE)

library(ggplot2)

go <- read.delim('enrich.go.txt', stringsAsFactors = FALSE)

go$term <- paste(go$ID, go$Description, sep = ': ')

go <- go[order(go$ONTOLOGY, go$p.adjust, decreasing = c(TRUE, TRUE)), ]

go$term <- factor(go$term, levels = go$term)

ggplot(go, aes(term, -log10(p.adjust))) +

geom_col(aes(fill = ONTOLOGY), width = 0.5, show.legend = FALSE) +

scale_fill_manual(values = c('#D06660', '#5AAD36', '#6C85F5')) +

facet_grid(ONTOLOGY~., scale = 'free_y', space = 'free_y') +

theme(panel.grid = element_blank(), panel.background = element_rect(color = 'black', fill = 'transparent')) +

scale_y_continuous(expand = expansion(mult = c(0, 0.1))) +

coord_flip() +

labs(x = '', y = '-Log10 P-Value\n')

write.table(enrich.go, 'enrich.go.txt', sep = '\t', row.names = FALSE, quote = FALSE)

####CIBERSORT#####

library(e1071)

library(parallel)

library(preprocessCore)

library(tidyverse)

library(ggplot2)

library(tinyarray)

library(tidyverse)

exp <- read.table("exp.txt",sep = "\t",row.names = 1,check.names = F,stringsAsFactors = F,header = T)

View(head(exp))

exp1 = rownames_to_column(exp)

write.table(exp1,file = "exp1.txt",row.names = F,quote = F,sep = "\t")

exp2 = exp1

source("CIBERSORT.R")

sig_matrix <- "LM22.txt"

mixture_file = 'exp1.txt'

res_cibersort <- CIBERSORT(sig_matrix, mixture_file, perm=1000, QN=TRUE)

save(res_cibersort,file = "res_cibersort.Rdata")

TME.results <- res_cibersort

TME.results[1:4,1:4]

re <- TME.results[,-(23:25)]

library(pheatmap)

k <- apply(re,2,function(x) {sum(x == 0) < nrow(TME.results)/2})

table(k)

re2 <- as.data.frame(t(re[,k]))

####plot#####

group <- read.csv("groups.csv",row.names = 1)

an = data.frame(group = group,

row.names = colnames(exp))

pheatmap(re2,scale = "row",

show_colnames = F,

annotation_col = an,

color = colorRampPalette(c("navy", "white", "firebrick3"))(50))

dev.off()

library(RColorBrewer)

mypalette <- colorRampPalette(brewer.pal(8,"Set1"))

dat <- re %>% as.data.frame() %>%

rownames_to_column("Sample") %>%

gather(key = Cell_type,value = Proportion,-Sample)

ggplot(dat,aes(Sample,Proportion,fill = Cell_type)) +

geom_bar(stat = "identity") +

labs(fill = "Cell Type",x = "",y = "Estiamted Proportion") +

theme_bw() +

theme(axis.text.x = element_blank(),

axis.ticks.x = element_blank(),

legend.position = "right") +

scale_y_continuous(expand = c(0.01,0)) +

scale_fill_manual(values = mypalette(22))

ggplot(dat,aes(Cell_type,Proportion,fill = Cell_type)) +

geom_boxplot(outlier.shape = 21,color = "black") +

theme_bw() +

labs(x = "Cell Type", y = "Estimated Proportion") +

theme(axis.text.x = element_blank(),

axis.ticks.x = element_blank(),

legend.position = "bottom") +

scale_fill_manual(values = mypalette(22))

a = dat %>%

group_by(Cell_type) %>%

summarise(m = median(Proportion)) %>%

arrange(desc(m)) %>%

pull(Cell_type)

dat$Cell_type = factor(dat$Cell_type,levels = a)

ggplot(dat,aes(Cell_type,Proportion,fill = Cell_type)) +

geom_boxplot(outlier.shape = 21,color = "black") +

theme_bw() +

labs(x = "Cell Type", y = "Estimated Proportion") +

theme(axis.text.x = element_blank(),

axis.ticks.x = element_blank(),

legend.position = "bottom") +

scale_fill_manual(values = mypalette(22))

dat <- dat %>% inner_join(groups,by="Sample")

library(ggpubr)

ggplot(dat,aes(Cell_type,Proportion,fill = Group)) +

geom_boxplot(outlier.shape = 21,color = "black") +

theme_bw() +

labs(x = "Cell Type", y = "Estimated Proportion") +

theme(legend.position = "top") +

theme(axis.text.x = element_text(angle=80,vjust = 0.5,size = 12))+

scale_fill_manual(values = mypalette(22)[c(6,1)])+ stat_compare_means(aes(group = Group,label = ..p.signif..),method = "kruskal.test")

ggboxplot(

dat,

x = "Cell_type",

y = "Proportion",

color = "black",

fill = "Group",

xlab = "",

ylab = "Cell composition",

) +

stat_compare_means(

aes(group = Group),

label = "p.signif",

method = "wilcox.test",

hide.ns = T,

size = 6

) +

scale_fill_manual(values=c("control" = "#4DBBD5", "IAs" = "#E64B35")

) +

theme(axis.text.x = element_text(

angle = 70,

hjust = 1,

vjust = 1

))

ggplot(data = dat, aes(x = Cell_type, y = Proportion, fill = Group)) +

geom_boxplot()+

stat_compare_means(aes(label = paste0("p = ", ..p.format..)), label.x = 1.35, vjust = 0.8, size = 4.5) +

guides(fill = FALSE)+

labs(x = "",

y = "Cell composition fraction")+

facet_wrap("Cell_type", scales = "free", nrow = 3)+

theme(axis.title=element_text(face = 'bold',size=12),

axis.text = element_text(size = 12, colour = 'black'),

axis.ticks.length=unit(.2, "cm"),

axis.ticks = element_line(colour = "black"),

panel.background = element_blank(),

panel.grid.major = element_blank(),

panel.grid.minor = element_blank(),

axis.line = element_line(colour = "black"),

panel.border = element_rect(colour = "black",size = 0, fill=NA),

plot.margin = margin(1, 1, 1, 1, "cm"))

library(tinyarray)

immu_data <- read.table("CIBERSORT-Results.txt",sep = "\t",row.names = 1,check.names = F,stringsAsFactors = F,header = T)

immu_data <- immu_data[-c(1:10),]

exp <- read.table("exp.txt",sep = "\t",row.names = 1,check.names = F,stringsAsFactors = F,header = T)

exp = t(exp)

expr_data <- exp

expr_data <- expr_data[-c(1:10),]

plot_df <- cbind(immu_data, expr_data)

###ggcorplot####

ggcorplot <- function(a,b,method="spearman"){

corr_eqn <- function(x,y,digits=3) {

test <- cor.test(x,y,method=method,exact=FALSE)

paste(paste0("n = ",length(x)),

paste0("r = ",round(test$estimate,digits),"(",method,")"),

paste0("p.value= ",round(test$p.value,digits)),

sep = ", ")

}

plot_df <- plot_df[,c(a,b)]

names(plot_df) <- c("ITGAX","COL4A2")

require(ggplot2)

ggplot(plot_df,aes(geneA,geneB))+

geom_point(col="black")+

geom_smooth(method=lm, se=T,na.rm=T, fullrange=T,size=2,col="red")+

geom_rug(col="#006fbc")+

theme_minimal()+

xlab(paste0(a," relative expression"))+

ylab(paste0(b," relative score"))+

labs(title = corr_eqn(plot_df$geneA,plot_df$geneB))+

theme(plot.title = element_text(hjust = 0.5),

plot.margin = margin(1, 1, 1, 1, "cm"))

}

yourgene = 'ITGAX'

ggcorplot(yourgene, colnames(immu_data)[1], method = 'spearman')

picDir <- './cor_picture/'

if (!dir.exists(picDir)) {

dir.create(picDir)

}

yourgene = 'COL4A2'

for (i in colnames(immu_data)) {

print(i)

inputgene = yourgene

p = ggcorplot(yourgene, i, method = 'spearman')

ggsave(p, filename = paste0(picDir, inputgene, '_',i,'_correlation', '.pdf'), width = 7, height = 5)

}

####WGCNA####

library(WGCNA)

library(reshape2)

library(stringr)

options(stringsAsFactors = FALSE)

enableWGCNAThreads()

exprMat <- "WGCNA/LiverFemaleClean.txt"

type = "unsigned"

corType = "pearson"

corFnc = ifelse(corType=="pearson", cor, bicor)

maxPOutliers = ifelse(corType=="pearson",1,0.05)

robustY = ifelse(corType=="pearson",T,F)

dataExpr <- read.table(exprMat, sep='\t', row.names=1, header=T,

quote="", comment="", check.names=F)

dim(dataExpr)

head(dataExpr)[,1:8]

m.mad <- apply(dataExpr,1,mad)

dataExprVar <- dataExpr[which(m.mad >

max(quantile(m.mad, probs=seq(0, 1, 0.25))[2],0.01)),]

dataExpr <- as.data.frame(t(dataExprVar))

gsg = goodSamplesGenes(dataExpr, verbose = 3)

if (!gsg$allOK){

# Optionally, print the gene and sample names that were removed:

if (sum(!gsg$goodGenes)>0)

printFlush(paste("Removing genes:",

paste(names(dataExpr)[!gsg$goodGenes], collapse = ",")));

if (sum(!gsg$goodSamples)>0)

printFlush(paste("Removing samples:",

paste(rownames(dataExpr)[!gsg$goodSamples], collapse = ",")));

# Remove the offending genes and samples from the data:

dataExpr = dataExpr[gsg$goodSamples, gsg$goodGenes]

}

nGenes = ncol(dataExpr)

nSamples = nrow(dataExpr)

dim(dataExpr)

head(dataExpr)[,1:8]

sampleTree = hclust(dist(dataExpr), method = "average")

plot(sampleTree, main = "Sample clustering to detect outliers", sub="", xlab="")

powers = c(c(1:10), seq(from = 12, to=30, by=2))

sft = pickSoftThreshold(dataExpr, powerVector=powers,

networkType=type, verbose=5)

par(mfrow = c(1,2))

cex1 = 0.9

plot(sft$fitIndices[,1], -sign(sft$fitIndices[,3])*sft$fitIndices[,2],

xlab="Soft Threshold (power)",

ylab="Scale Free Topology Model Fit,signed R^2",type="n",

main = paste("Scale independence"))

text(sft$fitIndices[,1], -sign(sft$fitIndices[,3])*sft$fitIndices[,2],

labels=powers,cex=cex1,col="red")

abline(h=0.85,col="red")

plot(sft$fitIndices[,1], sft$fitIndices[,5],

xlab="Soft Threshold (power)",ylab="Mean Connectivity", type="n",

main = paste("Mean connectivity"))

text(sft$fitIndices[,1], sft$fitIndices[,5], labels=powers,

cex=cex1, col="red")

power = sft$powerEstimate

power

net = blockwiseModules(dataExpr, power = power, maxBlockSize = nGenes,

TOMType = type, minModuleSize = 30,

reassignThreshold = 0, mergeCutHeight = 0.25,

numericLabels = TRUE, pamRespectsDendro = FALSE,

saveTOMs=TRUE, corType = corType,

maxPOutliers=maxPOutliers, loadTOMs=TRUE,

saveTOMFileBase = paste0(exprMat, ".tom"),

verbose = 3)

table(net$colors)

moduleLabels = net$colors

moduleColors = labels2colors(moduleLabels)

plotDendroAndColors(net$dendrograms[[1]], moduleColors[net$blockGenes[[1]]],

"Module colors",

dendroLabels = FALSE, hang = 0.03,

addGuide = TRUE, guideHang = 0.05)

MEs = net$MEs

MEs_col = MEs

colnames(MEs_col) = paste0("ME", labels2colors(

as.numeric(str_replace_all(colnames(MEs),"ME",""))))

MEs_col = orderMEs(MEs_col)

plotEigengeneNetworks(MEs_col, "Eigengene adjacency heatmap",

marDendro = c(3,3,2,4),

marHeatmap = c(3,4,2,2), plotDendrograms = T,

xLabelsAngle = 90)

load(net$TOMFiles[1], verbose=T)

TOM <- as.matrix(TOM)

dissTOM = 1-TOM

# Transform dissTOM with a power to make moderately strong

# connections more visible in the heatmap

plotTOM = dissTOM^7

# Set diagonal to NA for a nicer plot

diag(plotTOM) = NA

# Call the plot function

TOMplot(plotTOM, net$dendrograms, moduleColors,

main = "Network heatmap plot, all genes")

probes = colnames(dataExpr)

dimnames(TOM) <- list(probes, probes)

cyt = exportNetworkToCytoscape(TOM,

edgeFile = paste(exprMat, ".edges.txt", sep=""),

nodeFile = paste(exprMat, ".nodes.txt", sep=""),

weighted = TRUE, threshold = 0,

nodeNames = probes, nodeAttr = moduleColors)

trait <- "WGCNA/TraitsClean.txt"

if(trait != "") {

traitData <- read.table(file=trait, sep='\t', header=T, row.names=1,

check.names=FALSE, comment='',quote="")

sampleName = rownames(dataExpr)

traitData = traitData[match(sampleName, rownames(traitData)), ]

}

if (corType=="pearsoon") {

modTraitCor = cor(MEs_col, traitData, use = "p")

modTraitP = corPvalueStudent(modTraitCor, nSamples)

} else {

modTraitCorP = bicorAndPvalue(MEs_col, traitData, robustY=robustY)

modTraitCor = modTraitCorP$bicor

modTraitP = modTraitCorP$p

}

textMatrix = paste(signif(modTraitCor, 2), "\n(", signif(modTraitP, 1), ")", sep = "")

dim(textMatrix) = dim(modTraitCor)

labeledHeatmap(Matrix = modTraitCor, xLabels = colnames(traitData),

yLabels = colnames(MEs_col),

cex.lab = 0.5,

ySymbols = colnames(MEs_col), colorLabels = FALSE,

colors = blueWhiteRed(50),

textMatrix = textMatrix, setStdMargins = FALSE,

cex.text = 0.5, zlim = c(-1,1),

main = paste("Module-trait relationships"))
